# A Generalized Interpolation Material Point Method for Shallow Ice Shelves. 1: Shallow Shelf Approximation and Ice Thickness Evolution

**DOI:** 10.1029/2020MS002277

**Published:** 2021-08-24

**Authors:** Alex Huth, Ravindra Duddu, Ben Smith

**Affiliations:** ^1^ Department of Earth and Space Sciences University of Washington Seattle WA USA; ^2^ Now at Atmospheric and Oceanic Sciences Princeton University Princeton NJ USA; ^3^ Department of Civil and Environmental Engineering Vanderbilt University Nashville TN USA; ^4^ Department of Earth and Environmental Sciences Vanderbilt University Nashville TN USA; ^5^ Applied Physics Laboratory Polar Science Center University of Washington Seattle WA USA

**Keywords:** ice shelves, material point method, particle method, glaciology, damage, fracture

## Abstract

We develop a generalized interpolation material point method (GIMPM) for the shallow shelf approximation (SSA) of ice flow. The GIMPM, which can be viewed as a particle version of the finite element method, is used here to solve the shallow shelf approximations of the momentum balance and ice thickness evolution equations. We introduce novel numerical schemes for particle splitting and integration at domain boundaries to accurately simulate the spreading of an ice shelf. The advantages of the proposed GIMPM‐SSA framework include efficient advection of history or internal state variables without diffusion errors, automated tracking of the ice front and grounding line at sub‐element scales, and a weak formulation based on well‐established conventions of the finite element method with minimal additional computational cost. We demonstrate the numerical accuracy and stability of the GIMPM using 1‐D and 2‐D benchmark examples. We also compare the accuracy of the GIMPM with the standard material point method (sMPM) and a reweighted form of the sMPM. We find that the grid‐crossing error is very severe for SSA simulations with the sMPM, whereas the GIMPM successfully mitigates this error. While the grid‐crossing error can be reasonably reduced in the sMPM by implementing a simple material point reweighting scheme, this approach it not as accurate as the GIMPM. Thus, we illustrate that the GIMPM‐SSA framework is viable for the simulation of ice sheet‐shelf evolution and enables boundary tracking and error‐free advection of history or state variables, such as ice thickness or damage.

## Key Points

- Our material point method for ice shelf flow enables error‐free advection of history variables, such as damage, and ice front tracking
- The method can be readily implemented into existing finite element software (here, Elmer/Ice), and is suitable for large‐scale application
- The method is verified against analytical solutions for steady‐state flow and front evolution, and tested on an idealized marine ice sheet

## 1 Introduction

The fate of the entire Antarctic Ice Sheet is largely controlled by ice shelf dynamics. Over 80% of the Antarctic Ice Sheet drains into the ocean through floating ice shelves (Pritchard et al., 2012), where nearly all mass loss of the ice sheet occurs due to roughly equal contributions from basal melting and tabular calving (Depoorter et al., 2013; Paolo et al., 2015; Rignot et al., 2013). However, ice shelves also provide resistance to the flow of upstream grounded ice into the ocean, which primarily arises from contact with the walls of the bays in which they typically reside and localized grounding at pinning points such as ice rises and ice rumples. Any loss of this resistance, or buttressing, results in an increased flux of grounded ice flow into the ocean, thereby contributing to sea level rise (Dupont & Alley, 2005).

On decade to century timescales, the magnitude of ice shelf buttressing is controlled by ice front evolution (i.e., fluctuations in contact with bay walls/pinning points), fracture or thermomechanical weakening (e.g., Borstad et al., 2013; Sun et al., 2017), changes in ice thickness such as thinning from ocean‐driven basal melt (Cook et al., 2016; Pritchard et al., 2012), and response of the upstream grounded ice that feeds the shelf. Ideally, these four processes should be represented in a fully coupled manner that accounts for the complex feedbacks between them. For example, ice shelf thinning from basal melt has been associated with increased fracture (Liu et al., 2015; Shepherd et al., 2003), and fracture determines the ice front position through tabular calving. Calved icebergs can then alter local ocean properties and circulation within the ice shelf cavity and wherever they drift, which in turn, may affect basal melting rates (e.g., Cougnon et al., 2017; Robinson et al., 2010; Stern et al., 2015, 2016). Further, a more general motivation for developing an integrated representation of these processes stems from the lack of basal friction in ice shelves, which causes a highly nonlocal stress regime where altering stress in one part of the shelf can affect stresses throughout the shelf (Cuffey & Paterson, 2010). Therefore, it is important that we develop advanced numerical models and methods to enable realistic simulation of these processes controlling large‐scale ice shelf evolution, and thus gain a better understanding of Antarctic Ice Sheet dynamics and improve projections of sea level rise.

Current large‐scale ice flow models have difficulty in capturing the simultaneous processes of front evolution, fracture, and thinning owing to the differences in the modeling frameworks that are effective at describing each process separately. Because large‐scale ice flow is associated with extreme deformations, it is typically modeled within an Eulerian framework, where velocity is calculated as the ice flows through a fixed region in space. Typically, Eulerian models calculate flow velocity on a fixed mesh over time. However, some processes such as ice mass transport or fracture (represented by damage), are not well‐suited to the Eulerian approach due to the artificial diffusion or dispersion inherent to Eulerian advection schemes. For example, this artificial or numerical diffusion smears sharp edges and therefore compromises the accuracy of damage advection and evolution. Furthermore, Eulerian approaches require separate schemes to approximate ice front evolution, such as level‐set (Bondzio et al., 2016) or volume of fluid methods (Albrecht et al., 2011; Jouvet et al., 2008). In contrast, a Lagrangian approach, where the position of mesh nodes update with flow, avoids numerical diffusion and naturally tracks ice front evolution. However, Lagrangian or updated Lagrangian methods are only well‐suited for small deformation ice flow, such as within 2‐D flow‐band models for the propagation of individual crevasses over short timescales (Duddu & Waisman, 2013; Duddu et al., 2013, 2020; Jiménez et al., 2017; Sun et al., 2021). Use of Lagrangian methods to model entire ice shelf‐sheet systems could result in mesh degradation or tangling owing to the large deformations. Simple remeshing schemes are not ideal because they also introduce artificial diffusion.

These limitations of traditional Eulerian and Lagrangian schemes may be overcome using material point methods, which are formulated in a hybrid Eulerian‐Lagrangian framework that simultaneously allows large deformation flow, error‐free advection of history variables, and boundary tracking. The material point method (MPM) was originally introduced by Sulsky et al. (1994, 1995) for solid mechanics, as an adaptation of the particle‐in‐cell (PIC; Evans & Harlow, 1957) and fluid‐implicit‐particle (FLIP; Brackbill et al., 1988) methods. Henceforth, we will refer to this original version as the standard MPM (sMPM). In the sMPM, the material domain is discretized into a set of material points, or particles, that provide a Lagrangian description. Each material point has a mass, volume, position, velocity, stress, and any history variables or other material properties of the constitutive model. A background Eulerian mesh/grid is also defined, which extends beyond the initial domain defined by the material points, and typically remains fixed throughout the simulation. Grid cells containing material points constitute the “active” mesh on which the equations of motion are solved in a similar manner as the finite element method (FEM), but with material points serving as moving integration points. The mesh solution is then used to update material point variables and positions.

Many variants of the sMPM have been formulated that retain the basic procedure, but exhibit higher accuracy. These variants are largely motivated by the need to mitigate the well‐known “cell‐crossing error” in sMPM. This error arises from mapping between the material point and the background grid using linear shape functions, which have discontinuous gradients between grid cells so that abrupt transfers of stiffness occur as material points cross cell boundaries or become unevenly distributed between neighboring cells. The first sMPM variant to mitigate this error was the generalized interpolation material point method (GIMPM) developed by Bardenhagen and Kober (2004), which convolves the linear nodal shape functions with characteristic functions associated with each material point to result in continuous gradients between grid cells (see Figure S1). Other common variants of sMPM that modify the shape functions to have continuous gradients include the convected particle domain interpolation (CPDI) methods (Sadeghirad et al., 2011, 2013) and dual‐domain material point (DDMP) methods (Zhang et al., 2011). These variants of the sMPM have found diverse applications for modeling impact, fracture, and granular media behavior; for a more detailed literature review we refer the reader to Dunatunga and Kamrin (2015) and Coombs et al. (2020). Material point methods have also been used to model certain components of the cryosphere, including sea ice dynamics (Sulsky et al., 2007), snow (Stomakhin et al., 2013), and avalanches (Gaume et al., 2018).

Here, we develop an implementation of the GIMPM for simulating shallow‐shelf ice flow. To our knowledge, this is the first ever implementation of MPMs for shallow ice flow. Our GIMPM formulation solves the momentum and mass balance equations for ice flow and thickness evolution, and enables natural tracking of the ice front and grounding line. In Part II (Huth et al., 2021), we incorporate an anisotropic nonlocal creep damage model (Duddu & Waisman, 2012; Pralong & Funk, 2005) for fracture propagation. This paper solely focuses on the description and verification of the GIMPM in simulating shallow ice flow, ice thickness evolution, and ice‐ocean boundary treatment. We solve for ice flow velocities using the Shallow Shelf Approximation, or Shelfy‐Stream Approximation (SSA), a 2‐D vertically integrated flow model that is appropriate for large‐scale ice shelf and ice stream flow, where horizontal velocities can be considered vertically invariant (Macayeal, 1989). The SSA constitutes the only equations solved using the background Eulerian mesh/grid, while history variables such as ice thickness and damage are updated explicitly and efficiently on each material point directly. The primary advantage of our GIMPM formulation is that advection of all variables only involves updating the material point positions, thus our Lagrangian advection scheme is computationally inexpensive and avoids the artificial diffusion errors associated with Eulerian schemes. Furthermore, the positions of the material points allow us to establish and track the ice front and grounding line at sub‐grid scales. We implemented our model within the open‐source finite element ice flow model Elmer/Ice (Gagliardini et al., 2013), by modifying the Elmer SSA solver to implement GIMPM integration schemes and by introducing several modules for tracking and evolving the set of material points.

In the following sections, we will detail the derivation of our method and quantify its accuracy and stability for 1‐D and 2‐D ice flow simulations, including front advection. We will illustrate that the GIMPM‐SSA formulation is effective for: advecting history or internal state variables without diffusion, maintaining the steady‐state grounding lines of marine ice sheets, and tracking ice front evolution on century timescales. To ensure numerical accuracy, we formulate novel schemes for enforcing the conditions at the ice front and outflow boundaries, as well as for determining ice thickness at material points due to particle splitting. This paper is organized as follows: in Section 2 we review the SSA equations and their numerical discretization using the FEM and the GIMPM; in Section 3 we provide the details of our numerical implementation related to grid and particle variable updates; in Section 4 we present schemes for boundary treatment and error control; in Section 5 we provide examples that test the accuracy and numerical performance of the GIMPM‐SSA formulation; in Section 6 we provide a brief discussion on the pros and cons of the GIMPM, and finally, in Section 7 we make a few concluding remarks.

## 2 Governing Equations

In this section, we will briefly describe the governing equations of ice flow based on the Shallow Shelf Approximation (SSA), followed by the numerical discretization using the finite element and generalized material point methods. We will use indicial notation for vectors and tensors to describe the strong and weak forms of the governing equations and use matrix notation to present the corresponding discretized linear system. We will use Einstein's summation convention only for spatial indices, where repeated indices imply summation. For brevity, we occasionally avoid indicial notation, and use bold face letters to denote vectors, tensors and matrices.

### 2.1 Shallow Shelf Approximation

Ice shelves and ice streams can be modeled under the assumption of plug flow, where horizontal velocities and strain rates are constant over depth. Consequently, the incompressible Stokes equations are modified to exclude vertical shear and vertically integrated to derive the SSA that describes the horizontal force or momentum balance as

(1) $$\frac{\partial T_{ij}}{\partial x_{j}}+\;{\left(\tau_{b}\right)}_{i}=\;\rho gH\frac{\partial s}{\partial x_{i}}\;,$$

where spatial indices $i,j\in \left\{1,2\right\}$ correspond to the horizontal plane, $x=x_{i}{\hat{e}}_{i}$ denotes the in‐plane spatial coordinates, ${\hat{e}}_{i}$ are the basis vectors for the Cartesian coordinate system, ρ is the ice density, g is the acceleration due to gravity, H is the ice thickness, s is the top surface elevation, $\bar{\eta }$ is the depth‐averaged effective viscosity, and $\tau_{b}$ is the basal traction described by a friction law. For simplicity, here we assume the friction law as

(2) $${\left(\tau_{b}\right)}_{i}=\hat{\beta }v_{i}\;,$$

where $\hat{\beta }$ is a friction parameter and $v_{i}$ are the horizontal velocities for which the SSA is solved. Note that the above equation can used to specify several friction laws currently available in Elmer/Ice (Gagliardini et al., 2013) by defining $\hat{\beta }$ to be dependent on velocity, and in some cases, pressure. In Equation 1, the two‐dimensional vertically integrated stress tensor T is defined as (Bueler & Brown, 2009; Morland, 1987):

(3) $$T=2\bar{\eta }H\left[\begin{array}{ll} 2\frac{\partial v_{1}}{\partial x_{1}}+\frac{\partial v_{2}}{\partial x_{2}} & \frac{1}{2}\left(\frac{\partial v_{1}}{\partial x_{2}}+\frac{\partial v_{2}}{\partial x_{1}}\right)\\ \frac{1}{2}\left(\frac{\partial v_{1}}{\partial x_{2}}+\frac{\partial v_{2}}{\partial x_{1}}\right) & \frac{\partial v_{1}}{\partial x_{1}}+2\frac{\partial v_{2}}{\partial x_{2}} \end{array}\right],$$

which may alternatively be expressed in terms of strain rate ${\dot{\varepsilon }}_{ij}=1/2\left(\partial v_{i}/\partial x_{j}+\partial v_{j}/\partial x_{i}\right)$ as

(4) $$T=2\bar{\eta }H[\begin{array}{ll} 2{\dot{\varepsilon }}_{11}+\;{\dot{\varepsilon }}_{22} & {\dot{\varepsilon }}_{12}\\ {\dot{\varepsilon }}_{21} & {\dot{\varepsilon }}_{11}+2{\dot{\varepsilon }}_{22} \end{array}]\;.$$

The constitutive relation for ice flow relates deviatoric stress, $\sigma_{ij}^{D}$, to strain rate as

(5) $$\sigma_{ij}^{D}=\;2\eta {\dot{\varepsilon }}_{ij},$$

where the effective viscosity,η, follows the Norton‐Hoff flow law (Glen, 1955; Nye, 1957):

(6) $$\eta =\frac{1}{2}\;B{\dot{\varepsilon }}_{e}^{(1-n)/n}\;.$$

In the above equation, n is the flow law exponent, ${\dot{\varepsilon }}_{e}$ is the scalar second invariant or effective strain rate ${\dot{\varepsilon }}_{e}=\sqrt{{\dot{\varepsilon }}_{ij}{\dot{\varepsilon }}_{ji}/2}$, and B is a flow rate factor dependent on temperature and ice fabric. The depth‐averaged effective viscosity used in the SSA takes the same form as (Equation 6), but uses only in‐plane strain components to determine ${\dot{\varepsilon }}_{e}$ and a depth‐averaged rate factor, $\bar{B}=\;1/H\left(\int_{b}^{s}Bdz\right)$, where b is the vertical coordinate of the ice basal surface. We take z as positive in the upward direction, where z=0 corresponds to sea level.

A boundary condition at the ice front is set according to the seawater pressure at the ice terminus opposing ice flow

(7) $$\sigma_{ij}{\hat{n}}_{j}=\left\{\begin{array}{cc} \rho_{w}gz\;{\hat{n}}_{i} & \text{for}\;z<0\\ 0 & \text{for}\;z\geq 0 \end{array}\right.,$$

where $\sigma_{ij}=\sigma_{ij}^{D}-p\delta_{ij}$ is the Cauchy stress, p is the hydrostatic pressure, $\delta_{ij}$ is the Kronecker's delta, and $\hat{n}$ is the unit (outward) normal to the ice front. Equation 7 is depth‐integrated for implementation into the SSA as (Morland & Zainuddin, 1987):

(8) $$\;\int_{b}^{s}\sigma_{ij}{\hat{n}}_{j}dz=-\frac{1}{2}\rho_{w}gb^{2}{\hat{n}}_{i}.$$

Appropriate Dirichlet conditions for velocity are set at all other boundaries.

### 2.2 Weak Form and Discretization Using the FEM

The procedure for deriving the weak form of the SSA and discretization using the sMPM or the GIMPM is similar to that using the FEM, so we briefly review the procedure using the FEM first for clarity. Full details of this procedure can be found in the literature (e.g., Greve & Blatter, 2009; Lipscomb et al., 2019; Weis, 2001). The weak form of the SSA is derived using the Bubnov‐Galerkin method of weighted residuals by multiplying Equation 1 by an arbitrary smooth test function w(x) and integrating over the domain. After applying the divergence theorem and introducing the boundary conditions, we obtain:

(9) $$\begin{array}{l} \int_{\Omega }T_{ij}\frac{\begin{array}{l} \partial w_{i} \end{array}}{\partial x_{j}}d\Omega +\int_{\Omega }w_{i}\rho gH\frac{\partial s}{\partial x_{i}}d\Omega -\int_{\Omega }w_{i}{\left(\tau_{b}\right)}_{i}d\Omega \\ -\int_{\Gamma_{k}}w_{i}T_{ij}{\hat{n}}_{j}d\Gamma -\int_{\Gamma_{cf}}\frac{1}{2}w_{i}\left(\rho gH^{2}-\rho_{w}gb^{2}\right){\hat{n}}_{i}d\Gamma =0\;, \end{array}$$

where Ω and $\Gamma_{\text{cf}}$ represent the area of the ice domain and calving front boundary, respectively. Dirichlet conditions for velocity are applied along the final boundary, represented by $\Gamma_{k}.$ Here, $w_{i}$ is set to zero in accordance with the fixed velocities, and the integral over $\Gamma_{k}$ is eliminated (Macayeal, 1989).

All variables in Equation 9, including the test function w, are represented continuously on the mesh/grid using nodal shape functions. For example, velocity at a spatial location x and time t is defined as

(10) $$v\left(x,t\right)=\sum_{I=1}^{N_{n}}v_{I}\left(t\right)\phi_{I}\left(x\right),$$

where the nodes of the mesh are $x_{I},I=1,\ldots ,N_{n}$, $\phi_{I}\left(x\right)$ is the nodal shape function associated with node I, and $N_{n}$ is the number of nodes of the chosen finite element (here we use 4‐noded quadrilateral elements, so $N_{n}=4$). Substituting the continuous representations for v and w from Equation 10 into Equation 9, and noting that the test functions are arbitrary, a linear system can be assembled and solved for horizontal velocities v. The element tangent stiffness matrix K and residual force vector f can be split into components and expressed as follows:

(11) $$[\begin{array}{cc} K_{11} & K_{12}\\ K_{21} & K_{22} \end{array}][\begin{array}{c} v_{1}\\ v_{2} \end{array}]=[\begin{array}{c} f_{1}\\ f_{2} \end{array}],$$

where $v_{1}$ and $v_{2}$ are the vectors of nodal velocity components, the vectors $f_{1}$ and $f_{2}$ contain the gravitational forcing, and the element submatrices of the tangent matrix are given by

(12) $$\begin{array}{c} K_{11IJ}\;:=\underset{\Omega^{E}}{\int }2\bar{\eta }H\left(2\frac{\partial \phi_{I}\left(x\right)}{\partial x_{1}}\frac{\partial \phi_{J}\left(x\right)}{\partial x_{1}}+\frac{1}{2}\frac{\partial \phi_{I}\left(x\right)}{\partial x_{2}}\frac{\partial \phi_{J}\left(x\right)}{\partial x_{2}}\right)\mathrm{d\Omega}\\ +\int_{\Omega^{E}}\hat{\beta }\phi_{I}\left(x\right)\phi_{J}\left(x\right)\mathrm{d\Omega},\\ K_{22IJ}:=\int_{\Omega^{E}}2\bar{\eta }H\left(2\frac{\partial \phi_{I}\left(x\right)}{\partial x_{2}}\frac{\partial \phi_{J}\left(x\right)}{\partial x_{2}}+\frac{1}{2}\frac{\partial \phi_{I}\left(x\right)}{\partial x_{1}}\frac{\partial \phi_{J}\left(x\right)}{\partial x_{1}}\right)\mathrm{d\Omega}\\ +\int_{\Omega^{E}}\hat{\beta }\phi_{I}\left(x\right)\phi_{J}\left(x\right)\mathrm{d\Omega}\;,\\ K_{12IJ}:=\int_{\Omega^{E}}2\bar{\eta }H\left(\frac{\partial \phi_{I}(x)}{\partial x_{2}}\frac{\partial \phi_{J}(x)}{\partial x_{1}}+\frac{1}{2}\frac{\partial \phi_{I}(x)}{\partial x_{1}}\frac{\partial \phi_{J}(x)}{\partial x_{2}}\right)\mathrm{d\Omega},\\ K_{21IJ}:=\int_{\Omega^{E}}2\bar{\eta }H\left(\frac{\partial \phi_{I}(x)}{\partial x_{1}}\frac{\partial \phi_{J}(x)}{\partial x_{2}}+\frac{1}{2}\frac{\partial \phi_{I}(x)}{\partial x_{2}}\frac{\partial \phi_{J}(x)}{\partial x_{1}}\right)\mathrm{d\Omega}\;, \end{array}$$

where I and J denote the nodal indices of the element and $\Omega^{E}$ is the domain of the element, as indicated by the superscript, ‘E’. The right‐hand side of Equation 11 is given as

(13) $$\begin{array}{l} f_{1I}:=\int_{\Omega^{E}}\phi_{I}\left(x\right)\rho gH\frac{\partial s}{\partial x_{1}}d\Omega ,\\ f_{2I}:=\int_{\Omega^{E}}\phi_{I}\left(x\right)\rho gH\frac{\partial s}{\partial x_{2}}d\Omega \;. \end{array}$$

At boundary elements for the calving front,

(14) $$f_{iI}=\;\int_{\Gamma_{cf}^{E}}\frac{1}{2}\phi_{I}\left(x\right)\left(\rho gH^{2}-\rho_{w}gb^{2}\right){\hat{n}}_{i}d\Gamma =0\;.$$

Following standard finite element procedure, the integrals in Equations (12), (13), (14) are evaluated using Gaussian quadrature. Variables H, ρ, b, B, and ∇v must be mapped to the Gauss points from the nodes, where B and ∇v are used to calculate the depth‐averaged effective viscosity $\bar{\eta }$.

### 2.3 Weak Form and Discretization Using the GIMPM

The original formulation of the GIMPM (Bardenhagen & Kober, 2004) was derived using the Petrov‐Galerkin method, wherein the test function w and the trial function v belong to different function spaces. In the GIMPM, each material point or particle is assigned with a particle characteristic function, $\;\chi_{p}$, that must satisfy partition of unity in the reference or undeformed configuration

(15) $$\sum_{p}\chi_{p}(x,\;t=0)=1\;\forall \;x,$$

Note that partition of unity is also a requirement for the element shape functions. We choose $\chi_{P}$ to be the commonly used “hat” function with value one within the material point domain $\Omega_{p}$ and zero outside as

(16) $$\chi_{p}(x)=\left\{\begin{array}{cc} 1, & \;\text{if}\;x\in \Omega_{p},\\ 0, & \text{otherwise.} \end{array}\right.$$

Note that if $\chi_{p}\left(x\right)$ is chosen as a Dirac delta function, then the sMPM is retrieved. We assign a rectangular domain to each material point over which $\chi_{p}$ is defined, which we will refer to as the GIMPM domain of a material point. To satisfy Equations 15 and 16, the initial material domain must be discretized into material points so that no gaps or overlapping with neighboring GIMPM domains occurs. The GIMPM requires using a regular background grid of rectangular elements. We perform the initial discretization by evenly subdividing the domain Ω into GIMPM domains $\Omega_{p}$ by introducing a specified number of material points for each active background grid cell. In this formulation, we will update the lengths of the GIMPM domains due to deformation (see Section 3), with the goal of maintaining partition of unity over time precisely, to the extent possible. The area associated with a material point, $A_{p}$, is then defined as

(17) $$A_{p}=\int_{\Omega_{p}}\chi_{p}(x)d\Omega \;,$$

where $\Omega_{p}$ is the support area of the particle characteristic function and Ω is the area of the overall ice domain. Most literature on material point methods generalizes the formulation to 3‐D by using volume $\left(V_{p}\right)$ rather than area $\left(A_{p}\right)$, but we use $A_{p}$ here because the SSA is inherently 2‐D.

The values of material point variables may be initialized by integrating properties of the continuum body against the particle characteristic functions. For example, the initial value of material point property, $h_{p}^{0}$, may be expressed as an area‐averaged form of the initial continuum field $h^{0}(x)$ as

(18) $$h_{p}^{0}=\;\frac{1}{A_{p}^{0}}\int_{\Omega^{0}}h^{0}(x)\chi_{p}(x)d\Omega^{0},$$

where superscript ‘0’ indicates the initial time step. The validity of Equation 18 is a consequence of the partition of unity. Consistently, at any future time step m, the particle characteristic functions may be used as a basis to represent the material property throughout the computational domain

(19) $$h^{m}\left(x\right)=\sum_{p=1}^{N_{p}}h_{p}^{m}\chi_{p}\left(x\right),$$

where $N_{p}$ is the number of material points in the domain. We use Equation 19 to formulate continuous representations of vertically integrated stress ($T_{ij}$), gravitational driving force $\left(\rho gH\left(\partial s/\partial x_{i}\right)\right)$, and basal traction ${\left(\tau_{b}\right)}_{i}$, which we substitute into Equation 9 so that the integrals over the area of the entire ice domain become sums of integrals over material points. Further substituting in the C^0^ continuous representations for w using Equation 10, and utilizing that the test functions are arbitrary, we obtain the weak form as

(20) $$\begin{array}{c} \sum_{p=1}^{N_{p}}{\left(T_{ij}\right)}_{p}\frac{\partial S_{Ip}}{\partial x_{j}}A_{p}+\sum_{p=1}^{N_{p}}S_{Ip}\rho_{p}gH_{p}{\left(\frac{\partial s}{\partial x_{i}}\right)}_{p}A_{p}-\sum_{p=1}^{N_{p}}S_{Ip}{\left[{\left(\tau_{b}\right)}_{i}\right]}_{p}A_{p}\\ -\int_{\Gamma_{k}}\phi_{I}T_{ij}{\hat{n}}_{j}d\Gamma -\int_{\Gamma_{\text{cf}}}\frac{1}{2}\phi_{I}\left(\rho gH^{2}-\rho_{w}gb^{2}\right){\hat{n}}_{j}d\Gamma =0\;,\; \end{array}$$

where $S_{Ip}$ are the GIMPM weighting functions corresponding to node I (of the fixed background Eulerian mesh) evaluated at a material point location as

(21) $$S_{Ip}=S_{I}\left(x_{p}\right)=\;\frac{1}{A_{p}}\int_{\Omega_{p}}\chi_{p}\left(x\right)\phi_{I}\left(x\right)d\Omega \;,$$

and with the gradient defined as

(22) $$\frac{\partial S_{Ip}}{\partial x_{i}}=\;\frac{1}{A_{p}}\int_{\Omega_{p}}\chi_{p}\left(x\right)\frac{\partial \phi_{I}\left(x\right)}{\partial x_{i}}d\Omega .$$

We take the nodal shape functions $\phi_{I}$ to be element‐wise linear Lagrange interpolants. These shape functions at the two nodes of the linear element can be defined as

(23) $$\begin{array}{l} \phi_{1}=\phi_{1}\left(x\right)=\frac{1-\xi }{2}\;,\\ \phi_{2}=\phi_{2}\left(x\right)=\frac{1+\xi }{2}\;, \end{array}$$

where $\xi \left(x\right)$ is the local (or isoparametric) coordinate (between −1 and +1) of any material or integration point within the parent element. For illustration, we plot both the linear and GIMPM shape functions in 1‐D in supporting information Figure S1. The GIMPM shape function exceeds the boundaries of a single element and is C^1^ continuous by smoothing the discontinuous gradient observed in the linear shape functions, which mitigates cell‐crossing error. Material points influence all elements that their GIMPM domains overlap. Note that in practice, we evaluate the convolutions in Equations 21 and 22 as the overlap of $\chi_{p}$ and the linear shape functions within each element rather than for each node individually, which allows us to assemble stiffness matrices is a similar manner to the FEM (see, e.g., Charlton et al., 2017). Additionally, we note that replacing the linear shape functions with higher order interpolants would be problematic as the latter are not positive throughout the element. Further substituting into Equation 20 the C^0^ continuous representations for v using Equation 10 yields element sub‐matrices that are computed by summing over material points:

(24) $$\begin{array}{l} K_{11IJ}\;:=\sum_{p=1}^{n_{p}}A_{p}2{\bar{\eta }}_{p}H_{p}\left(2\frac{\partial \phi_{Ip}}{\partial x_{1}}\frac{\partial S_{Jp}}{\partial x_{1}}+\frac{1}{2}\frac{\partial \phi_{Ip}}{\partial x_{2}}\frac{\partial S_{Jp}}{\partial x_{2}}\right)+\sum_{p=1}^{n_{p}}A_{p}{\hat{\beta }}_{p}\phi_{Ip}S_{Jp},\\ K_{22IJ}\;:=\sum_{p=1}^{n_{p}}A_{p}2{\bar{\eta }}_{p}H_{p}\left(2\frac{\partial \phi_{Ip}}{\partial x_{2}}\frac{\partial S_{Jp}}{\partial x_{2}}+\frac{1}{2}\frac{\partial \phi_{Ip}}{\partial x_{1}}\frac{\partial S_{Jp}}{\partial x_{1}}\right)+\sum_{p=1}^{n_{p}}A_{p}{\hat{\beta }}_{p}\phi_{Ip}S_{Jp\;},\\ K_{12IJ}\;:=\sum_{p=1}^{n_{p}}A_{p}2{\bar{\eta }}_{p}H_{p}\left(\frac{\partial \phi_{Ip}}{\partial x_{2}}\frac{\partial S_{Jp}}{\partial x_{1}}+\frac{1}{2}\frac{\partial \phi_{Ip}}{\partial x_{1}}\frac{\partial S_{Jp}}{\partial x_{2}}\right),\\ K_{21IJ}\;:=\sum_{p=1}^{n_{p}}A_{p}2{\bar{\eta }}_{p}H_{p}\left(\frac{\partial \phi_{Ip}}{\partial x_{1}}\frac{\partial S_{Jp}}{\partial x_{2}}+\frac{1}{2}\frac{\partial \phi_{Ip}}{\partial x_{2}}\frac{\partial S_{Jp}}{\partial x_{1}}\right), \end{array}$$

where $n_{p}$ is the number of material points in the element and we use the same shorthand for the linear shape functions evaluated at a material point location, $\phi_{Ip}$ = $\phi_{I}\left(x_{p}\right)$, as defined for the GIMPM functions in Equation 21. Similarly, the components of the body force vector are computed as

(25) $$\begin{array}{l} f_{1I}:=\sum_{p=1}^{n_{p}}S_{Ip}\rho_{p}gH_{p}{\left(\frac{\partial s}{\partial x_{1}}\right)}_{p}A_{p},\\ f_{2I}:=\sum_{p=1}^{n_{p}}S_{Ip}\rho_{p}gH_{p}{\left(\frac{\partial s}{\partial x_{2}}\right)}_{p}A_{p}. \end{array}$$

Comparing Equations 24 and 25 with Equations 12 and 13, we can notice the subtle differences between the GIMPM and the standard FEM. We address the numerical treatment of the ice front boundary for the GIMPM/sMPM in Section 4.1.

By replacing $S_{Jp}$ and $\partial S_{Jp}/\partial x_{i}$ with $\phi_{Jp}$ and $\partial \phi_{Jp}/\partial x_{i}$, respectively, in Equations (24), (25) we can obtain the sMPM. However, errors in the sMPM can accumulate if the sum of material point area (weights) within an element significantly varies from the element area or if the distribution of material points within the element becomes irregular. In Section 5, we show how increasing the number of material points can mitigate this error to a certain extent, but ultimately, an alternative material point weighting is required to more evenly distribute material point weights between elements and maintain accuracy (Gonzalez Acosta et al., 2017). The new weight, $W_{p}$, is a function of both the element area, $A_{E}$, and material point areas, $A_{p}$, as given by

(26) $$W_{p}=\;\frac{A_{E}}{\sum_{p=1}^{n_{p}}A_{p}}\;.$$

Thus, in the reweighted sMPM, $W_{p}$ replaces $A_{p}$ as the integration weights in Equations 24 and 25. This reweighting can also be used with the GIMPM, where $A_{p}$ becomes the area of overlap between a GIMPM domain and the element. However, reweighted GIMPM is mostly unnecessary because material point weight is already smoothly distributed between neighboring elements unless severe overlaps or gaps develop between neighboring GIMPM domains. We largely avoid these errors in our simulation studies, and therefore do not apply the reweighting to our GIMPM simulations here.

## 3 Numerical Implementation

**Figure 1:**
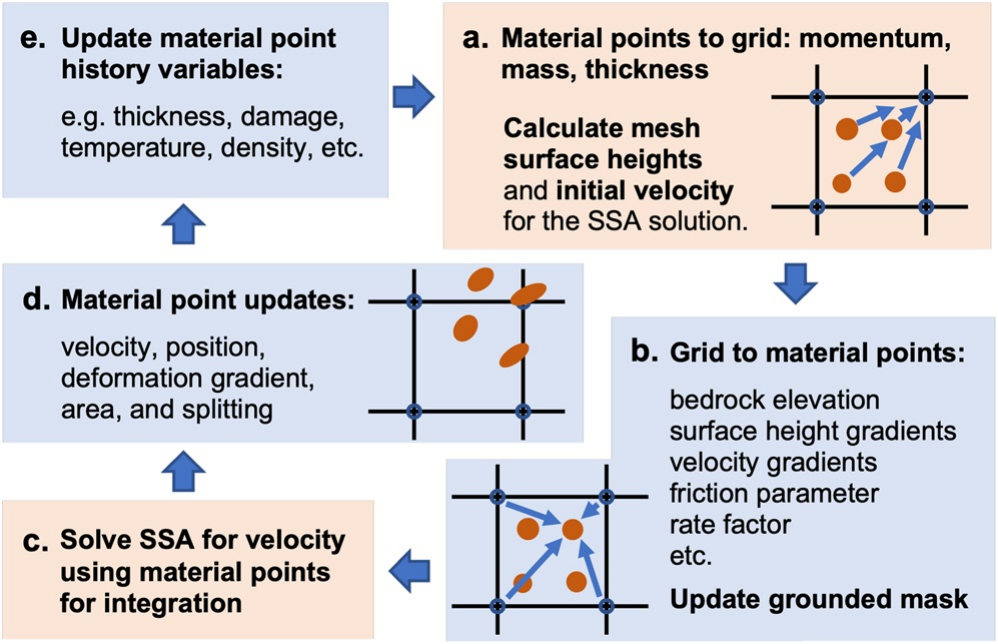
Material point method‐Shallow Shelf Approximation (MPM‐SSA) numerical procedure. The orange dots are material points and blue circles are grid nodes. Grid processes (Eulerian) are highlighted in red and material point processes (Lagrangian) are highlighted in blue.

At time t=0, the ice domain is discretized into a specified number of material points per grid cell as described in Section 2.2. The unknown variables, namely ice flow velocity and ice thickness, are defined directly on the material points; whereas, the external parameters such as bedrock elevation, the basal friction parameter, and accumulation/ablation rates are defined on the background fixed mesh. For simplicity, each simulation presented here uses a constant flow rate factor B and density ρ for all material points. However, these quantities can be treated as spatially varying and history‐dependent. In this section, we detail the numerical procedure for a typical computational cycle, according to the simplified representation given in Figure 1. The cycle begins with a series of parameter mappings between the material points and the grid (Figures 1a and 1b), which are needed in preparation for solving the SSA. The mappings are used to initialize the SSA grid velocity and to determine all parameters at the material point level needed to compute Equations 24 and 25. The SSA is solved using an iterative routine (Figure 1c), where material point viscosity is updated alongside grid velocity until convergence. Subsequently, the grid solution is used to update material point positions, velocities, and geometric parameters (Figure 1d). Finally, material point history variables are updated (Figure 1e), which only includes ice thickness in this study. To improve readability, we will use matrix notation for vectors and tensors to avoid showing spatial indices and show only node and particles indices to explain the mapping between nodes and particles.

### 3.1 SSA Initialization: Grid Parameters

To allow a solution of the SSA, the velocity field and thickness are initialized on the grid by mapping from the material points (Figure 1a), where the thickness on the grid is subsequently converted to surface elevation. The gradients of surface elevation and velocity are mapped to material points for the SSA matrix assembly. The initialized grid velocity is further required as part of the update routine for material point velocity (Section 3.4). The velocity mapping from particles to nodes is performed using a formula that enforces momentum conservation:

(27) $$v_{I}=\frac{\sum_{p}^{N_{p}}m_{p}v_{p}S_{Ip}}{\sum_{p}^{N_{p}}m_{p}S_{Ip}},$$

where $m_{p}$ is the material point mass

(28) $$m_{p}=\rho_{p}H_{p}A_{p}\;.$$

Ice thickness is mapped to the grid from material points as

(29) $$H_{I}=\frac{\sum_{p}^{N_{p}}H_{p}S_{Ip}A_{p}}{\sum_{p}^{N_{p}}S_{Ip}A_{p}},$$

where the denominator is necessary to normalize the interpolation. After each mapping, nodal values of velocity or thickness at Dirichlet boundaries are overwritten with the values specified by the essential boundary condition. Nodal surface elevations are calculated from the nodal ice thicknesses as

(30) $$s_{I}=b_{I}+H_{I}\;,$$

where the nodal elevation of the ice base, $b_{I}$, is computed as the maximum value of the bedrock elevation ($z_{\text{bed}}$) or the ice base elevation according to hydrostatic equilibrium as

(31) $$b_{I}=\text{max}\left\{{\left(z_{\text{bed}}\right)}_{I},\;z_{\text{sea}}-H_{I}\left(\frac{\rho }{\rho_{w}}\right)\right\}\;,$$

and $z_{\text{sea}}=0$ is the sea level.

### 3.2 SSA Initialization: Material Point Parameters

The second half of the SSA initialization procedure is focused on updating material point variables (Figure 1b). Surface height and velocity gradients are determined at any material point p by mapping from the nodes. The friction parameter $\left({\hat{\beta }}_{p}\right)$, bedrock elevation $\left({z_{\text{bed}}}_{p}\right)$, and rate factor ($B_{p}$) must also be defined at the material point level, which may require mapping from the nodes as well. Any scalar grid property, $h_{I}$, may be interpolated to the material points as

(32) $$h_{p}=\sum_{I}^{N_{n}}h_{I}S_{Ip}.$$

Similarly, for gradients, the mapping is

(33) $$\nabla h_{p}=\sum_{I}^{N_{n}}h_{I}\nabla S_{Ip}.$$

Lastly, material points are marked as grounded or floating. Defining grounding status at material points (or at Gauss points in the FEM) rather than at nodes during the SSA solution has been shown to provide a more accurate estimate of grounding line dynamics during the SSA solution (Seroussi et al., 2014). However, to be consistent with Elmer/Ice conventions, we also define grounding status at the nodal level as part of the procedure to define the sub‐element scale grounding line. If node I has $b_{I}={\left(z_{\text{bed}}\right)}_{I}$, it is marked as grounded; otherwise, it is floating. If a material point belongs to an element whose surrounding elements have a mix of grounded and floating nodes, then that it is clear that material point is near the grounding line, and its grounding status is determined using the same procedure used for the nodes. Otherwise, it inherits the grounding status of its surrounding nodes.

### 3.3 SSA Solution

The SSA is solved implicitly using an “iteration on viscosity” scheme where we update material point viscosity, ${\bar{\eta }}_{p}$, each iteration until convergence (Macayeal, 1989; Figure 1c). This is done by mapping the gradients of nodal velocity solution from the previous iteration to material points using Equation 33, which are converted to strain‐rates to calculate ${\bar{\eta }}_{p}$ using Equation 6. We achieve quick convergence of the SSA solution using the Biconjugate Gradient Stabilized (BiCGSTAB) method, Incomplete LU preconditioning, and a combination of Picard and Newton iterations.

### 3.4 Material Point Updates

Upon completion of the SSA, grid velocities are used to update material point velocities, position, and geometric properties (Figure 1d).

*Velocities and position*: To update velocity and position, material point methods typically adopt the approach of the FLIP method. For velocity, this update is given as

(34) $$v_{p}^{m+1}=v_{p}^{m}+\Delta t\sum_{I}^{N_{n}}a_{I}^{m}S_{Ip}=\;v_{p}^{m}+\sum_{I}^{N_{n}}(v_{I}^{m+1}-v_{I}^{m})S_{Ip},$$

where $a_{I}^{m}$ is the acceleration at time step m at node I, and $v_{I}^{m}$ is the nodal velocity previously interpolated to the grid from the material points before the SSA solution in Equation 27. This material point position update is

(35) $$x_{p}^{m+1}=x_{p}^{m}+\Delta t\overset{N_{n}}{\underset{I}{\sum }}v_{I}^{m+1}S_{Ip}\;.$$

In practice, the FLIP update scheme can introduce noise that results from the mismatch between the number of material points and grid nodes, so our code also includes the update scheme XPIC (k), an algorithm that can remove FLIP noise using a set of k additional projections (Hammerquist & Nairn, 2017). Lower orders of k may introduce undesired damping, while higher orders of k are computationally expensive. Note that the additional projections required for XPIC(k) can accumulate a small amount of error in conjunction with our boundary treatment at the ice front (Section 4.1), but this error can be avoided by always using FLIP updates within k elements of the ice front. While the simulations in this paper are relatively insensitive to the update scheme chosen, Nairn et al. (2017) demonstrated that XPIC(5) yields sharp and stable crack propagation in damage simulations.

*Geometric properties*: All updates to material point geometric properties, which include area and the lengths defining the GIMPM domain, depend on the deformation gradient, a fundamental kinematic quantity that characterizes the deformation at a material point based on its current (deformed) and reference (undeformed) spatial coordinates. The material point deformation gradients ($F_{p}$) are tracked over time, and are updated as

(36) $${F_{p}}^{m+1}=\left(I+\Delta t\nabla v_{p}^{m+1}\right){F_{p}}^{m}\;.$$

where I is the second‐order identity tensor. In the sMPM, the determinant of $F_{p}$ is used to update the material point area as

(37) $${A_{p}}^{m+1}=\text{det}\left({F_{p}}^{m+1}\right)A_{p}^{0}\;.$$

In the GIMPM, material point area is calculated as the product of the lengths defining the rectangular GIMPM domain.

Our implementation currently includes two schemes to update GIMPM domain lengths. The lengths should be updated carefully in order to minimize overlap or separation of GIMPM domains over time, and thus maintain partition of unity as precisely as possible throughout the domain. The first scheme updates the lengths of a GIMPM domain such that the resulting rectangular domain approximates the quadrilateral domain that would be obtained if the position of each corner of the GIMPM domain was updated individually (Coombs et al., 2020). In practice, this “corner‐tracking” update scheme may be simplified to tracking the midpoints ${\hat{x}}_{p}$ of the GIMPM domain edges as

(38) $${\hat{x}}_{p}={\hat{x}}_{p}^{m}+\Delta t\underset{I}{\sum }v_{I}^{m+1}\phi_{I}\left({\hat{x}}_{p}^{m}\right)\;.$$

The GIMPM domain lengths can be obtained using the maximum and minimum extents of ${\hat{x}}_{p}$ as

(39) $${\left(l_{p}^{m+1}\right)}_{i}=\frac{1}{2}\left[\text{max}{\left({\hat{x}}_{p}\right)}_{i}-\text{min}{\left({\hat{x}}_{p}\right)}_{i}\right]\;,$$

followed by a correction that guarantees proper volume (area in 2‐D) as

(40) lpm+1i=lpm+1idetFklm+1∏j=1nDlp0j∏j=1nDlpm+1j1nD.

where $n_{D}$ is the dimension of the problem ($n_{D}=2$ in our case). More detailed derivations of the above scheme can be found in Coombs et al. (2020). This corner‐tracking scheme performs well in minimizing overlap or separation of GIMPM domains over time in any flow regime, but cannot be used at outflow boundaries where a GIMPM domain may only partially overlap the active background grid, assuming velocities beyond the active grid are unknown (see Section 4.1). For these material points, we instead use the second update scheme, given by

(41) $${\left(l_{p}^{m+1}\right)}_{i}={\left(l_{p}^{0}\right)}_{i}{U_{ii}}^{m+1}\;\left(no\;implied\;sum\;on\;i\right),$$

where ${\left(l_{p}^{0}\right)}_{i}$ are the original domain lengths and $U_{ij}=\sqrt{{F_{ki}}^{T}F_{kj\;}}$ is the symmetric material stretch tensor, which is equivalent to the deformation gradient rotated into the original Cartesian reference frame (Charlton et al., 2017). Although the “stretch‐tensor” update scheme can be used instead of the “corner‐tracking” scheme in the entire domain, we caution that it is less capable of minimizing overlap or separation of GIMPM domains under large shearing deformation. Because the “stretch‐tensor” scheme is sufficiently accurate under stretching and rotation and is computationally more efficient than the “corner‐tracking” scheme, the former scheme is suitable for simulations without large shearing deformations. Here, we only use the stretch‐tensor scheme for the 1‐D flow‐band simulations, noting that the corner‐tracking scheme gives identical results. In all the 2‐D simulations, we employ the corner‐tracking scheme.

### 3.5 History Variable Updates

The computational cycle finishes by updating the history variables on the material points (Figure 1e). Here, we only consider ice thickness ($H_{p}$), which is updated explicitly according to the Lagrangian description of surface mass conservation for a column of ice at time step m+1 as

(42) $$H_{p}^{m+1}\;=H_{p}^{m}+({\dot{a}}_{p}^{m}\;-\;\nabla \cdot v_{p}^{m}H_{p}^{m})\Delta t\;,$$

where ${\dot{a}}_{p}^{m}$ (m a^−1^) is the sum of the basal and surface accumulation rates. We add damage as a history variable in Part II (Huth et al., 2021).

## 4 Boundary Treatment and Splitting

Boundary conditions in MPMs may be applied at the edges of the active computational grid as in the FEM. However, special treatment is required at inflow boundaries to properly introduce new material points to the domain, and at outflow boundaries where material points must be eliminated or GIMPM domains may partially overlap the boundary. Further treatment is also needed at the moving ice front boundary to avoid integration errors, as this boundary may not align with element edges. We detail our boundary treatment in this section. In addition, we detail our material point splitting scheme, which mitigates additional integration errors that may arise under tension, where the resolution of material points per grid cell decreases over time as the area of the material points grows.

### 4.1 Boundary Treatment

**Figure 2:**
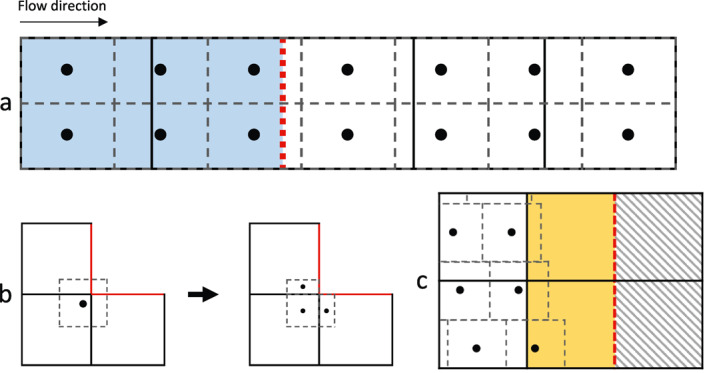
Material point method‐Shallow Shelf Approximation (MPM‐SSA) boundary treatment. (a) At inflow boundaries (dotted red), an additional domain (shaded blue) is specified upstream and seeded with extra material points (black dots). Each material point is associated with a generalized interpolation material point method (GIMPM) domain (dashed gray rectangles). Velocities are specified throughout the additional domain so that the extra material points advect into the primary domain at the correct velocity. (b) A material point with a GIMPM domain overlapping an outflow boundary (red) is split into sub‐particles during grid‐to‐material point mappings. The sub‐particles separately receive the interpolation, which is subsequently consolidated back to the original material point. (c) At the ice front, grid cells partially full with material points domains (yellow) are integrated using the finite element method (FEM), where the boundary condition is assigned at the element edges (dashed red) that mark the transition between active and inactive (gray‐striped) grid cells.

*Inflow boundaries*: Since material points at inflow boundaries advect downstream, a scheme is needed to ensure that that they are replaced by inflow of new material points. For the simple simulations in this paper, we incorporate inflow boundaries by seeding additional material points on a domain that extends beyond the boundaries. Velocities on the extra domain and inflow boundary are set so that the additional material points flow smoothly into the primary domain at the velocity specified by the boundary condition. This scheme is illustrated in Figure 2a, where the material point GIMPM domains are dotted gray, the inflow boundary is indicated by the dotted red line, and elements belonging to the additional inflow domain are highlighted in blue. We note that a more efficient, but complicated, scheme may be implemented, where in the “inflow elements” refill with material points automatically as they become empty (Zhao et al., 2019).

*Outflow boundaries*: At outflow boundaries, material points exit the domain and are removed from the simulation. In the GIMPM, material points with GIMPM domains that overlap an outflow boundary will not receive a full interpolation during grid to material point mappings by default, assuming parameter values are unknown beyond the active portion of the background grid. In Figure 2b (left side), a material point GIMPM domain is shown overlapping an outflow boundary (red). For each active element that the GIMPM domain overlaps, our treatment is to temporarily introduce a sub‐particle with a GIMPM domain matching the area of overlap between the original material point domain and the element (Figure 2b, right side). The sub‐particles receive the interpolation, with the original material point then receiving the average of sub‐particle values weighted by the area of their subdomains.

*Ice front boundary*: While the position of the ice front is naturally tracked by material points positions, it will rarely align with element edges. Applying the ice front stress boundary condition along element edges results in large integration errors if the element is not sufficiently full of material points. To mitigate this issue, we forgo material point integration for elements containing the ice front. Instead, we employ Gauss quadrature (i.e., the finite element method) within the element, which allows us to enforce the boundary condition at the element edge that divides active and inactive elements. An illustration of this treatment is shown in Figure 2c, where inactive elements are shaded with gray stripes, partially full ice front elements being approximated with the FEM are shaded with yellow, and the ice front boundary is indicated by the dashed red line. Note that using FEM at the ice front requires mapping the material point history variables used in the SSA solution to the element nodes in the same manner as Equation 29. We show in Section 5 that using the FEM at the ice front is sufficiently accurate; however, alternative solutions exist if desired. One approach would be to apply the boundary condition along line segments or B‐splines that approximate the sub‐element scale location of the ice front (Bing et al., 2019). If using the sMPM, an additional option would be to adjust or replace the mesh to always align with the position of the ice front, and maintain sMPM integration within the element and FEM boundary conventions. This treatment would be possible because sMPM is not restricted to a rectilinear grid.

### 4.2 Material Point Splitting

The highly tensile regime of ice shelves tends to cause material points to elongate or grow over time. Material points can be split as necessary to maintain a desired resolution of material points per grid cell. For the GIMPM, we initiate splitting when the domain length $l_{p}$ exceeds a given threshold. We implement a similar procedure for the sMPM, where a pseudo‐domain length is tracked using the accumulated strain of a material point in Cartesian directions (Ma et al., 2009). The splitting threshold cannot exceed the length of a grid cell, and can vary across the domain if, for example, greater material point resolution is desired near the grounding line. For splitting in direction i, the two split material point coordinates, xps1i and xps2i are set to

(43) xps1i=(xp)i+14(lp)i,xps2i=(xp)i−14(lp)i.

Each new material point is then assigned half the current ${\left(l_{p}^{m+1}\right)}_{i}$ and initial $(l_{p}^{0}){\;}_{i}$ domain length corresponding to the splitting direction i, from the parent material point being split. For a unidirectional split, the non‐split current and initial domain lengths are inherited from the parent material point without modification. The deformation gradient and velocities of the parent material point are transferred directly to the new material points, but direct transfer of thickness may cause visible thickness oscillations in areas of steep thickness gradients. We propose to mitigate these oscillations by instead reassigning thickness to each split material point as

**Figure 3:**
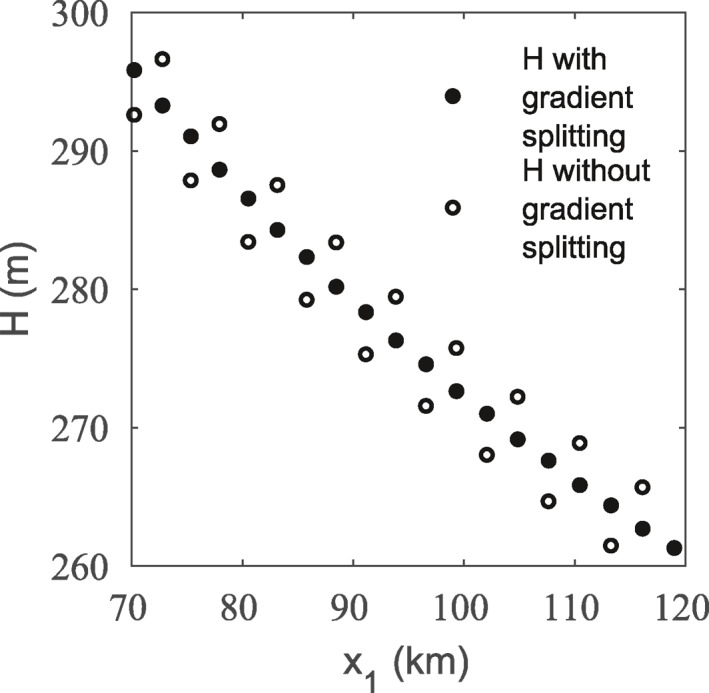
Thickness for a subset of material points at the end of the steady state flow‐band test with and without adjusting thickness according to its gradient during splitting. This simulation used the generalized interpolation material point method (GIMPM) at 5 km grid resolution and 4 material points per cell.

(44) hsp=Hp+∂Hp∂xi[(xp)is−(xp)i].

where the thickness gradient, $\partial H_{p}/\partial x_{i}$, must be interpolated from the grid. Figure 3 gives the thicknesses for a subset of material points at the end of the steady state flow‐band test described in Section 5 (GIMPM at 5 km grid resolution and 4 material points per cell), both with and without adjusting thickness according to Equation 44. By using Equation 44, the thickness oscillations from splitting are almost fully eliminated.

## 5 Examples

In this section, we consider several examples using the GIMPM and sMPM for SSA simulations to validate and test the methods. We quantify error in modeled stress and front propagation versus analytical solutions in 1‐D, and further demonstrate front propagation in 2‐D. We then test the methods on an idealized marine ice sheet to show that they can maintain steady state grounding line positions over time and can advect passive scalar fields without artificial diffusion.

### 5.1 Flow‐Band Test Case: Steady State

We test our GIMPM‐SSA framework against a flow‐band model that gives the analytical steady state for a longitudinally unconfined ice shelf with a constant flux at the upstream inflow boundary. The flow‐band model is formulated under the assumption of unidirectional flow, and is therefore inherently 1‐D. In practice, we model the flow‐band in 2‐D, where the domain is one element wide, but unidirectional flow is still enforced (i.e., ${\left(v_{2}\right)}_{p\;}=0$). This experiment was previously used to verify a finite‐difference front‐tracking scheme (Albrecht et al., 2011), and we use the same values for ice density (ρ=910 kg m^−3^), seawater density ($\rho_{w}=1028$ kg m^−3^), and the flow rate factor ($B=1.9\times 10^{8}$ Pa s^1/3^). The flux at the upstream boundary is given as $Q_{0}=v_{0}H_{0}$, where we take the velocity, $v_{0}=300$ m a^−1^ and the thickness, $H_{0}=600$ m. The solution for the spreading rate is given as

(45) $$\frac{\partial v_{1}}{\partial x_{1}}={\left(\frac{\rho g}{4B}\left(1-\frac{\rho }{\rho_{w}}\right)H\right)}^{3}=CH^{3}\;,$$

where all flow is along the $x_{1}$‐axis (Weertman, 1957). The analytical deviatoric stress can be calculated using Equations 5 and 6. The thickness and velocity profiles are obtained from conservation of mass and momentum are given by $H\left(x_{1}\right)=\;{\left(4C/Q_{o}x_{1}+1/H_{0}^{4}\right)}^{1/4}$ and $\;v_{1}\left(x_{1}\right)=Q_{0}/H\left(x_{1}\right)\;$, respectively (van der Veen, 2013).

**Figure 4:**
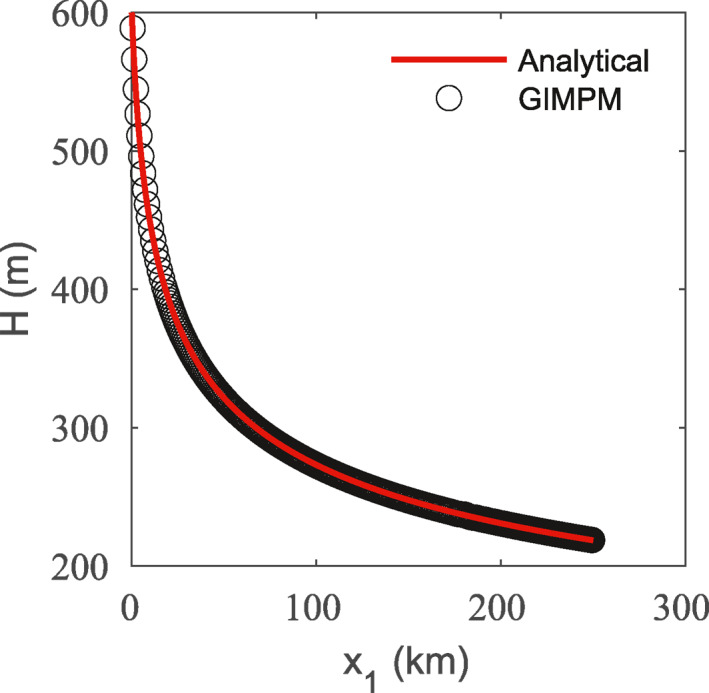
Analytical steady state ice thickness for the flow‐band test and the generalized interpolation material point method (GIMPM) solution at 300 years using a 2.5 km resolution grid and 9 material points per cell.

We first test the ability of the GIMPM‐SSA model to maintain the given steady state. We consider a domain that spans from the inflow boundary at $x_{1}\;=\;0$ to a fixed ice front at $x_{1}\;=\;250$ km. The steady‐state thickness corresponding to this configuration is shown in Figure 4. The initial material point locations fully cover this domain, as well an additional domain beyond the inflow boundary that must be included to enforce the inflow boundary condition. The initial velocity, $v_{0}$, and thickness, $H_{0}$, is always enforced on all nodes of the inflow domain. The first element of the primary domain, immediately adjacent to the inflow domain, is also given special treatment. We always enforce the analytical velocity and thickness on all nodes of this element, and we set the material point ice thicknesses within this element to the analytical solution. This inflow thickness and velocity correction scheme alleviates any error in material point ice thicknesses that would otherwise occur from the jump in velocity gradient between the inflow and primary domains.

Note that the analytical solution does not include an ice front, as $H\left(x_{1}\right)=0$, but including an ice front at any location on the domain will not change the steady‐state upstream provided the ice front boundary conditions (Equation 8) are assigned. Setting the ice front at $x_{1}\;=\;250$ km gives a realistic thickness at the ice front of ∼219 m. We test the sMPM and the GIMPM at varying material point resolutions, and with and without the reweighting given by Equation 26. We use a 2.5 km resolution grid. Each trial is initialized with the analytical solutions for thickness and velocity, and run forward for 300 years using one‐month time steps. The threshold material point length at which splitting is initiated is set to 1.5 times the original length. The length after splitting is then 0.75 times the original length, which due to the purely tensile flow regime, therefore constitutes the lower bound on potential lengths that will develop throughout the simulation.

**Figure 5:**
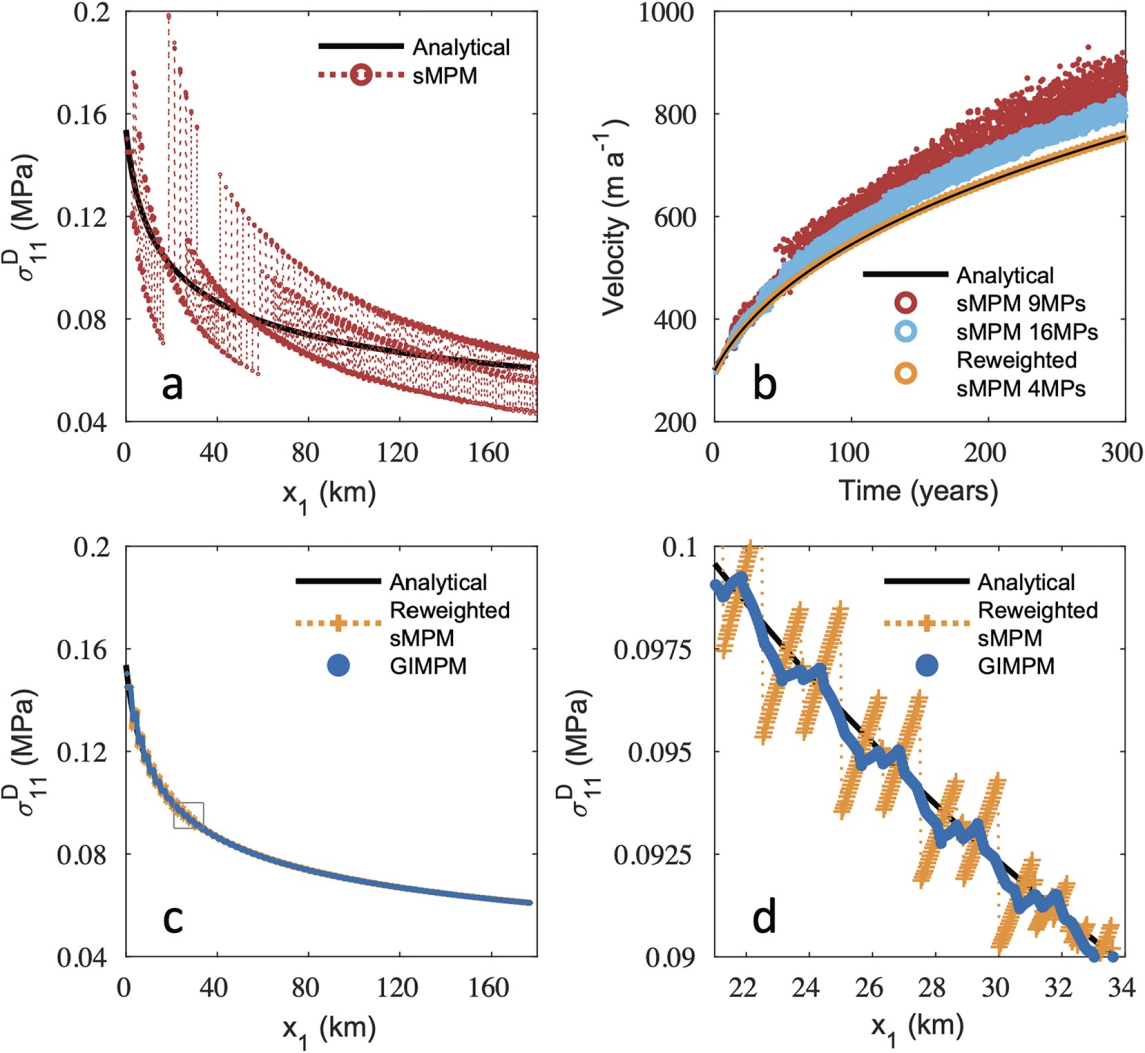
Results from the steady state flow‐band test for the material point initially located closest to 0 km, where 9 material points are initialized per 2.5 km grid cell. (a) Deviatoric stress using the unweighted standard material point method (sMPM). (b) Velocities corresponding to (a) compared to the velocities obtained using 16 material points per cell, as well as the velocities using the reweighted sMPM with only 4 material points per cell. (c) Deviatoric stress using the reweighted sMPM and the generalized interpolation material point method (GIMPM), which closely match the analytical result. (d) Detail of the boxed region in (c). The discontinuities for the reweighted sMPM are caused by the grid‐crossing error, and are largely alleviated using the GIMPM.

Figure 5 shows the deviatoric stress and velocities for the material point initially located closest to $x_{1}=0$ km as it advects to its final location of ∼177 km over 300 years. Unless otherwise indicated, these figures are initialized with 9 material points per cell (3×3 in 2‐D). Figure 5a compares the result that does not use the reweighting scheme from Equation 26 with the analytical result. Stresses fluctuate widely due to uneven material point weighting between elements, which results in inaccurate velocities, positions, and thicknesses. Figure 5b gives the velocities from the sMPM when using 9 and 16 material points per cell and the reweighted sMPM when using 4 material points per cell. It is evident that increasing the material point resolution in the sMPM may slightly mitigate the weighting error, but it increases the computational expense and is not nearly as accurate as reweighted sMPM. The reweighted sMPM ensures a smoother transition of the stiffness matrix between elements, and even with just 4 material points per cell, yields results that almost exactly match the analytical solution. The severity of the error without the reweighting scheme is not common to all MPM simulations, and is likely due to the highly nonlocal stress regime of the SSA. As there appears to be very little tolerance for this type of error, the reweighting scheme from Equation 26 appears to be essential for accurate SSA simulations using the sMPM.

The stress response using the reweighted sMPM and the GIMPM are given in Figure 5c, and show significant improvement over the sMPM in Figure 5a. Note that the reweighting scheme has no effect when implemented with the GIMPM, as no gaps or overlaps of the GIMPM domains develop in the test case. The fit with the analytical solution is less accurate where $x_{1}<∼\;40$ km, as ice shelf surface slopes are high and therefore finer mesh resolution is needed for improved accuracy. In general, the GIMPM is more accurate than the reweighted sMPM, as the latter still does not alleviate cell crossing errors as fully. This is evident in Figure 5d, which shows the zoom of the region within the gray box from Figure 5c. The GIMPM alleviates, but still cannot entirely eliminate the sharp stress discontinuities or oscillations as the material crosses cell boundaries.

**Figure 6:**
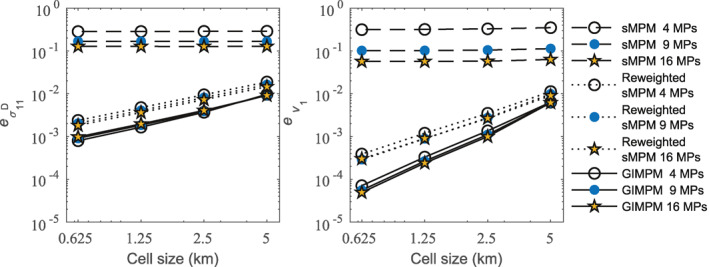
Error for horizontal deviatoric stress (left) and velocity (right) for the steady state flow‐band test.

We further investigate the performance of our methods by conducting a mesh convergence study. We run the steady‐state flow‐band model again using the GIMPM, sMPM, and reweighted sMPM, but now we test each method using four different grid resolutions: 5, 2.5, 1.25, and 0.625 km. We use a time step increment of 5 days and run each simulation for 150 years. For consistency between all grid resolutions, we also extend the inflow thickness and velocity correction scheme, applied previously to the first element of the primary domain, to include all elements in the primary domain with a downstream nodal coordinate of ${\left(x_{I}\right)}_{1}\leq 5$ km. For each grid and method, three material point resolutions are tested: 4, 9, and 16 material points per cell. Each time step, we compute the normalized errors (de Vaucorbeil et al., 2020)

$$e_{\sigma_{11}^{D}}=\sqrt{\frac{\sum_{m=1}^{m_{f}}\sum_{p=1}^{N_{p}}A_{p}^{m}{\left[\sigma_{11}^{D}{\left(x_{p}^{m}\right)}^{a}-\sigma_{11}^{D}{\left(x_{p}^{m}\right)}^{n}\right]}^{2}}{\sum_{m=1}^{m_{f}}\sum_{p=1}^{N_{p}}A_{p}^{m}{\left[\sigma_{11}^{D}{\left(x_{p}^{m}\right)}^{a}\right]}^{2}}},$$

(46) $$e_{v_{1}}=\sqrt{\frac{\sum_{m=1}^{m_{f}}\sum_{p=1}^{N_{p}}A_{p}^{m}{\left[v_{1}{\left(x_{p}^{m}\right)}^{a}-v_{1}{\left(x_{p}^{m}\right)}^{n}\right]}^{2}}{\sum_{m=1}^{m_{f}}\sum_{p=1}^{N_{p}}A_{p}^{m}{\left[v_{1}{\left(x_{p}^{m}\right)}^{a}\right]}^{2}}}$$

for horizontal deviatoric stress and velocity, respectively. In these equations, superscripts n and a indicate the numerical and analytical values, respectively, $m_{f}$ is the total number of time steps, and $N_{p}$ is the number of material points with ${\left(x_{p}\right)}_{1}\geq 5$ km. Figure 6 gives the error for the steady state flow‐band test for all combinations of methods, material point resolutions, and grid resolutions. The corresponding convergence rates averaged over all material point resolutions are given in Tables 1 and 2 for deviatoric stress and velocity, respectively.

**Table 1:** Deviatoric Stress Convergence Rates at Each Cell Size, and Over all Cell Sizes

Cell size (km)	sMPM	Reweighted sMPM	GIMPM
2.5 km	0.01	1.0	1.27
1.25 km	0.0	1.0	1.09
0.625 km	0.0	1.0	1.03
Overall	0.0	1.0	1.13

Abbreviations: GIMPM, generalized interpolation material point method; sMPM, standard material point method.

For large deformation that occurs over 150 years, Figure 6 and Tables 1 and 2 show that the (unweighted) sMPM is the least accurate of the three methods tested, and that it does not converge with mesh refinement. The poor performance of unweighted sMPM is due to grid‐crossing error, especially due to uneven material point weighting between elements; whereas, the reweighted form of the sMPM shows substantial improvements in accuracy and convergence. The reweighted sMPM has overall or average convergence rates of approximately 1 and 1.65 for deviatoric stress and velocity, respectively. These convergence rates are close to the theoretical convergence rates for linear FEM, which in this case are 1 for deviatoric stress and 2 for velocity (de Vaucorbeil et al., 2020). The GIMPM is the most accurate of the methods tested as it best alleviates grid‐crossing error. In agreement with previous studies (e.g., Charlton et al., 2017), GIMPM shows greater convergence rates than the theoretical linear FEM convergence rates, but less than the theoretical quadratic FEM convergence rates of 2 for deviatoric stress and 3 for velocity.

**Table 2:** Velocity Convergence Rates at Each Cell Size, and Over all Cell Sizes

Cell size (km)	sMPM	Reweighted sMPM	GIMPM
2.5 km	0.11	1.77	2.45
1.25 km	0.03	1.58	2.05
0.625 km	0.01	1.61	2.25
Overall	0.05	1.65	2.23

Abbreviations: GIMPM, generalized interpolation material point method; sMPM, standard material point method.

In Figure 6, only unweighted sMPM shows a significant decrease in error for both velocity and deviatoric stress when the number of material points are increased, because this alleviates grid‐crossing and improves the accuracy of integration to a limited extent. For reweighted sMPM and GIMPM, grid resolution has a much larger influence on accuracy than the number of material point used to integrate each grid cell, although both methods do show a slight decrease in velocity error as the number of material points is increased. For reweighted sMPM, there is also a slight decrease in deviatoric stress error with increased material point resolution, whereas the opposite trend is observed for the GIMPM for deviatoric stress error, due to greater smoothing of the stress discontinuity between grid cells (Bardenhagen & Kober, 2004).

### 5.2 Flow‐Band Test Case: Front Propagation

We also use the flow‐band model to test the ability of our scheme to track the calving front. The analytical position of the ice front, $x_{c}$, at time *t* can be found from the relation $Q_{0}t=\;\int_{0}^{x_{c}}H\left(x^{\prime }\right)dx^{\prime }$ (Albrecht et al., 2011), and is given by

(47) $$x_{c}\left(t\right)=\;\frac{Q_{0}}{4C}\left[{\left(3Ct+\frac{1}{H_{0}^{3}}\right)}^{\frac{4}{3}}-\frac{1}{H_{0}^{4}}\right]\;.$$

**Figure 7:**
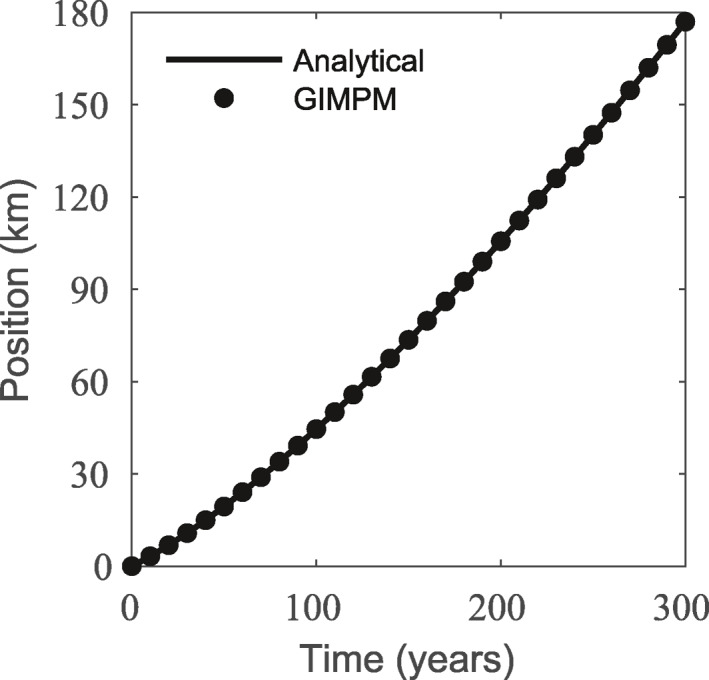
Ice front position using the generalized interpolation material point method (GIMPM) plotted against the analytical solution using 9 material points per 2.5 km grid cell. This nearly perfect fit is reflected for all grid and material point resolutions tested, for both the GIMPM and reweighted standard material point method (sMPM).

For all grid and material point resolutions from the mesh convergence exercise, and using the GIMPM and reweighted sMPM, we track the ice front over 300 years using one month time steps, where the initial position of the ice front is set to $x_{1}=0\;\text{km}$. The modeled front position over time using the GIMPM with a 2.5 km grid and 9 material points per grid cell is shown in Figure 7 versus the analytical solution. This nearly perfect fit is reflected for all grid and material point resolutions tested, for both the GIMPM and reweighted sMPM. After 300 years of front propagation, none of the simulations deviate from the analytical front position of $x_{1}\approx 177\;\text{km}\;$ by more than 300 m (∼0.17% error). Thus, this study demonstrates that the GIMPM can accurately simulate the stresses, geometry, and ice front position of an evolving ice shelf.

### 5.3 Front Advection in 2‐D

**Figure 8:**
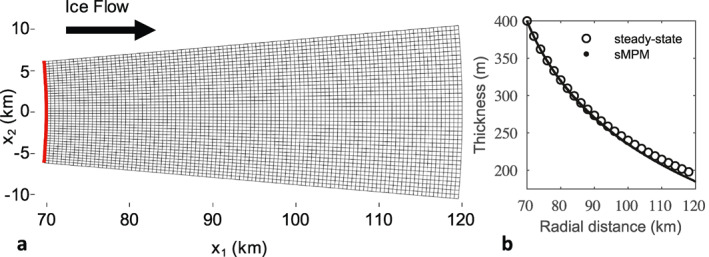
(a) Background grid used to simulate the unconstrained 2‐D spreading of an ice tongue from an upstream boundary (red) with a 70 km radius of curvature. (b) The ice thicknesses of all material points after growing the ice tongue from the upstream boundary for 86.6 years, and the steady‐state thickness profile calculated from the finite element method (FEM) using the same ice front position. Material point thicknesses are slightly lesser toward the ice front due to the increased rate of spreading these material points experienced earlier in the simulation, when the ice tongue was shorter and buttressing was lesser.

To test our front propagation scheme in 2‐D, we simulate the radial spread of an unconstrained floating ice tongue. This benchmark example was considered under steady‐state conditions in previous studies (e.g., Morland & Zainuddin, 1987; Pegler & Worster, 2012, 2013; Wearing et al., 2020). Our aim here is to achieve only qualitatively consistent results, because there is no analytical solution for 2‐D diverging ice flow. The setup of this the simulation follows Example 1 in Wearing et al. (2020). The grid is shown in Figure 8a, and is comprised of non‐uniform, linear, quadrilateral elements with straight edges. We use the reweighted sMPM, because the grid elements are not perfect rectilinear elements owing to the ice shelf geometry; whereas, the GIMPM using the hat function $\chi_{p}$ for the particle characteristic function requires the use of perfect rectilinear elements. However, the reweighted sMPM can be classified as a special case of the GIMPM that uses the Dirac delta function instead of the hat function for the particle characteristic function. The upstream boundary (red) corresponds to an arc extracted from a circle with 70 km radius with a central angle of 10°. Flow is axisymmetric with respect to the vertical axis defined at the center of the circle, and we set free slip conditions at the lateral boundaries by enforcing that the normal component of velocity is zero. At the upstream boundary, a constant thickness of 400 m and an inflow velocity of 500 m a^−1^ is enforced. We evenly initialize 9 material points per cell on an inflow domain beyond the upstream boundary (not shown), and allow the system to evolve until the ice front reaches the downstream edge of the computational grid, which occurs after 86.6 years.

The corresponding final thicknesses and positions of all material points are plotted in Figure 8b. The thicknesses of all material points at any radial distance match very closely regardless of their azimuthal position, reflecting that the simulation has achieved the expected axisymmetric flow regime. Also plotted is the steady‐state thickness profile as calculated using the FEM under the assumption that the calving front is fixed at the downstream edge. While it is encouraging that the two thickness profiles show similar trends, we emphasize that unlike in the 1‐D case, we do not expect a simulation with a moving ice front to replicate the steady state flow exactly. Some mismatch is expected because an unconstrained ice tongue experiences buttressing that increases proportionately with ice tongue length (Wearing et al., 2020). This buttressing is related to “hoop” stresses that must be overcome for flow to diverge laterally. In Figure 8b, the material points toward the ice front are relatively thin compared to the steady state because they endured larger rates of spreading earlier in the simulation when the ice tongue was short and buttressing was lesser. This example demonstrates that material point methods can be used for 2‐D ice front tracking in a physically consistent manner.

### 5.4 Marine Ice Sheet Model Intercomparison Project (MISMIP+)

**Figure 9:**
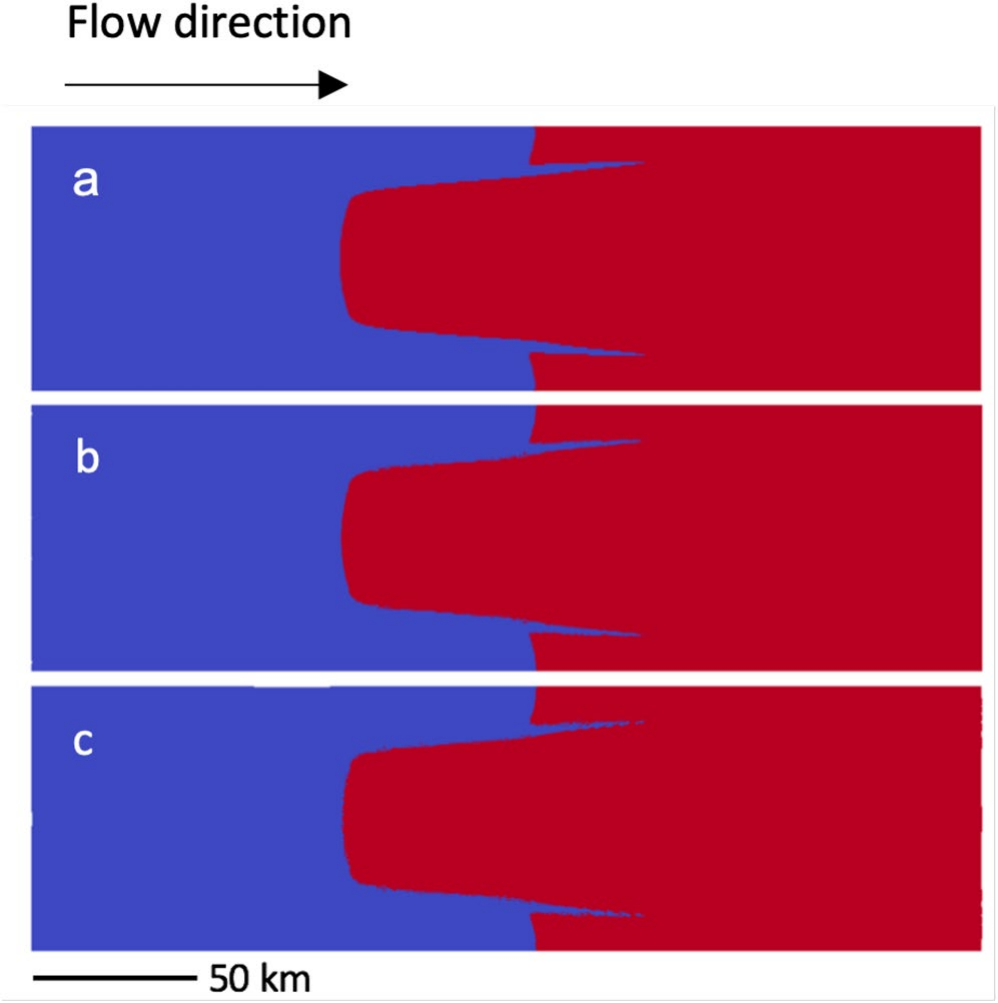
(a) Grounding line at 0 years after initiating the generalized interpolation material point method (GIMPM)/standard material point method (sMPM), where blue material points are grounded and red are floating. The configuration after 100 years is shown for (b) the GIMPM and (c) the reweighted sMPM.

Our final experiment tests the ability of our model to maintain the steady state from the idealized, but more realistic, geometry detailed in the marine ice sheet model intercomparison project (MISMIP+; Asay‐Davis et al., 2016). This geometry is a 640 km × 80 km marine ice sheet, spanning an ice divide at $x_{1}=0$ km to a calving front at 640 km. At steady state, the grounding line is centered at $x_{1}\;∼450$ km and $x_{2}=40$ km. At the lateral boundaries, $v_{2}=0.$ The steady state grounding configuration where $x_{1}\;>350$ km is shown in Figure 9a. The grounding line lies on a retrograde slope, and is therefore very sensitive to perturbations or error, so that the configuration is ideal for testing the accuracy of the GIMPM. Furthermore, this is a very high shear regime, which is often problematic for the MPMs. Therefore, we update GIMPM domains with the corner‐tracking scheme from Equations (38), (39), (40).

For all MISMIP + simulations, we use a grid resolution of 0.5 km and time step size of one month. We initially determine the steady state using the FEM according to the recommended values for the friction parameter, the viscosity parameter, the rate factor, densities, and surface accumulation given for the MISMIP+. Afterward, we continue the simulation using both the GIMPM and the reweighted sMPM. We initialize these simulations with 9 material points per cell. After 100 years, both the GIMPM (Figure 9b) and the reweighted sMPM (Figure 9c) are able to maintain the sensitive initial grounding line position (Figure 9a). The reweighted sMPM grounding line region, however, is slightly noisier than GIMPM. This noise is especially evident in a close‐up view of the image in Figure 9c (zoom not shown).

**Figure 10:**
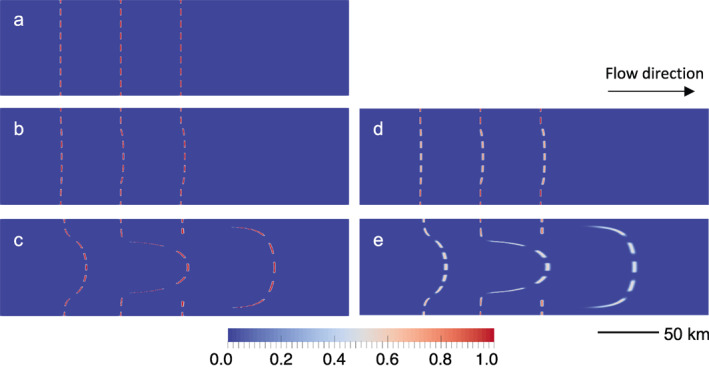
Marine ice sheet model intercomparison project (MISMIP+) advection of a passive scalar. Results shown for same area as Figure 9 around the grounding line for the generalized interpolation material point method (GIMPM) versus Discontinuous Galerkin (DG). The initial state is given in (a). The field at 5 years is shown for (b) the GIMPM and (d) DG. The field at 100 years is shown for (c) the GIMPM and (e) DG.

During the simulation, we also advect a passive scalar field to demonstrate how when using the GIMPM, this field can be advected without artificial diffusion. This field is initially assigned a value of unity along a series of across‐flow strips and a value of zero elsewhere (Figure 10a). Each strip is separated by 50 km in the $x_{1}$‐direction. Though the scalar is passive and does not affect flow, we loosely associate the scalar field with damage. We therefore chose the width of the strips to be 0.5 km to roughly correspond to the width of an ice shelf rift, which can range from zero to several kilometers wide. The field was initially assigned on the grid, and interpolated to the material points before the simulation. For comparison, we ran the same simulation using a Discontinuous Galerkin (DG) method (Brezzi et al., 2004), the least‐diffusive Eulerian advection method already available in Elmer/Ice. During the DG advection solution, we limit the value of the scalar to be non‐negative and no greater than 1, because we associate the scalar field with damage. This constraint is automatically satisfied with the GIMPM because the value of the scalar for a material point is never changed over time. However, in the DG method, these constraints must be enforced on the domain in Elmer using a scheme where they are applied and released iteratively according to a criterion based on residuals (Gagliardini et al., 2013). Whether or not we bound the scalar values using the constraint scheme, we notice that the same broad patterns of artificial diffusion are obtained with the DG method.

The advected profiles of the scalar field obtained from the GIMPM are shown in Figure 10b after 5 years and Figure 10c after 100 years. The scalar field does not experience artificial diffusion, and takes an arcuate shape over time that reflects the high shear experienced from the lateral grounded margins. The results from the DG method are shown in Figure 10d after 5 years and in Figure 10e after 100 years. Although the DG method produces a similar arcuate profile for the scalar field as the GIMPM, the value of the scalar field is diminished due to numerical diffusion over time. With the DG method, the furthest downstream, non‐zero values of the scalar near the centerline of the *y*‐domain (*y* = 40 km) quickly diminish from 1.0 to ∼0.8 over 5 years. By 100 years, the diffusion increases in severity, and these furthest‐downstream, non‐zero values diminish to ∼0.5. Consistently, the results from the DG method show the spatial spread of the damage region to be about twice that observed from the GIMPM results (Figure 10c). Thus, this simulation study illustrates the superior performance of the GIMPM, based on a hybrid Lagrangian‐Eulerian framework, in alleviating numerical diffusion issues persistent with the DG method in a purely Eulerian framework.

**Figure 11:**
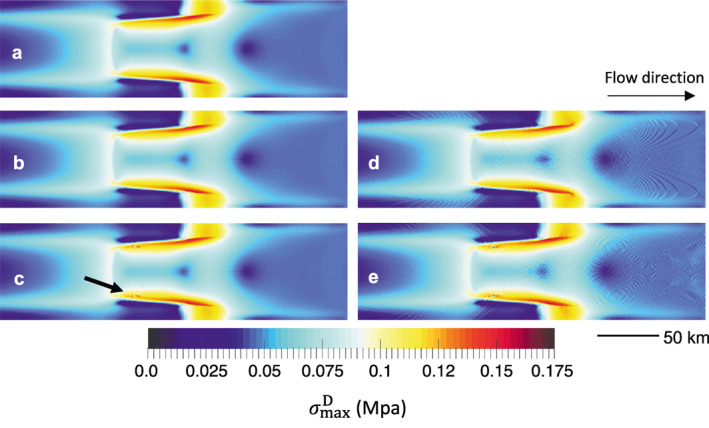
Marine ice sheet model intercomparison project (MISMIP+): Maximum principal deviatoric stresses (MPa) for the (a) initial state, (b) generalized interpolation material point method (GIMPM) at 50 years, (c) GIMPM at 100 years, (d) reweighted standard material point method (sMPM) at 50 years, (e) reweighted sMPM at 100 years. The arrow in (c) indicates where continual heavy shear eventually causes poorly distributed material points, as shown in detail in Figure 12.

**Figure 12:**
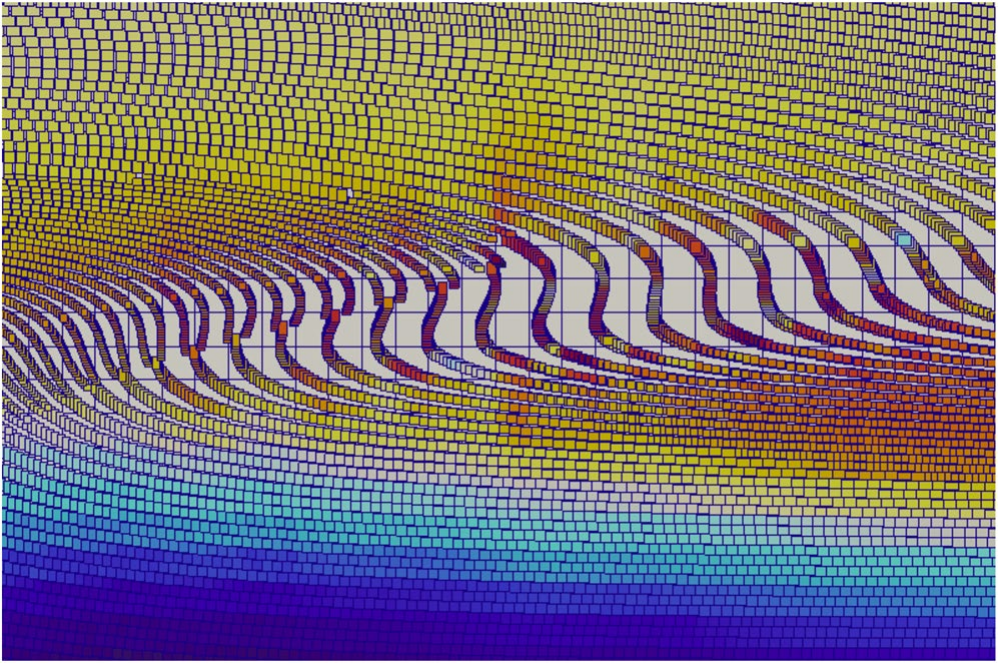
Marine ice sheet model intercomparison project (MISMIP+): Poorly distributed material points develop in the generalized interpolation material point method (GIMPM) simulation after 100 years of heavy shear, where indicated by the arrow in Figure 11c. Underlying grid resolution is 0.5 km.

Figure 11 shows the maximum principal deviatoric stress, $\sigma_{\text{max}}^{D}$, for the MISMIP + test obtained using the GIMPM and the reweighted sMPM after t = 100 years. The initial $\sigma_{\text{max}}^{D}$ is given in Figure 11a, where the largest stresses are concentrated near the lateral grounding line. The GIMPM field after 50 years (Figure 11b) is almost identical to the initial field. The reweighted sMPM field at 50 years (Figure 11d) while mostly identical to the initial field, however, is characterized by oscillations due to grid‐crossing error, which also cause the noise in the grounding line configuration in Figure 9c. By 100 years, both the GIMPM (Figure 11c) and the reweighted sMPM (Figure 11e) stress fields develop some artifacts in the stress field near the grounding line, as material points tend to become poorly distributed under extreme shear (Figure 12). This type of error is a limitation of our current GIMPM implementation; we discuss potential approaches to alleviate it in Section 6.

## 6 Discussion

Our current GIMPM or reweighted sMPM formulations should be sufficiently accurate for many applications in which it is essential to accurately track the ice front or history variables, such as damage (Huth et al., 2021). However, additional developments are needed to mitigate the artifacts introduced due to intense distortion of material point domains in high shear regimes over long timescales (Section 5.4). One approach is to reinitialize the material points periodically, which in the simplest case would involve interpolating all material point properties to a new set of material points. Although this approach risks some artificial diffusion, it may be negligible if reinitialization is infrequent. However, more sophisticated schemes are also available that reinitialize material points locally as needed, while minimizing artificial diffusion (e.g., Yue et al., 2015).

Further development of our method will likely include implementing more robust shape functions. For example, Convected Particle Domain Interpolation (CPDI) methods assemble shape functions according to the shapes of the material point domains, and alleviate cell‐crossing error. Unlike the GIMPM, CPDI methods are not restricted to tracking rectangular domains, and instead may track parallelograms (CPDI1; Sadeghirad et al., 2011) or the corners of the domains individually (CPDI2; Sadeghirad et al., 2013). The CDPI1 method has been shown to perform especially well under intense shearing (Wang et al., 2019), and may be appropriate for avoiding the errors related to high‐shear observed in our GIMPM simulations over long timescales. However, we note that our current GIMPM formulation is computationally less expensive and easier to implement into existing finite element codes. Implementing CPDI methods will require substantial modifications to our current discretization scheme and boundary treatment.

As an alternative to using material point domain‐tracking shape functions, it may also be advantageous to consider techniques that eliminate cell‐crossing error through other means, such as the dual domain material point (DDMP) method (Zhang et al., 2011) or the use of spline‐based shape functions (e.g., Stomakhin et al., 2013). All of these techniques, including CPDI, share an additional advantage over the GIMPM in that they may be employed using non‐uniform meshes of varying element types, such as triangular meshes commonly used in major ice flow codes (e.g., ISSM and Elmer/Ice). Analyzing the error, convergence qualities, and speed of these methods in the context of ice shelf flow and fracture will constitute future research.

Although not illustrated in this paper, an additional advantage of our GIMPM‐SSA model is that complex 3‐D multiphysics can be represented while still being efficient enough to couple with Earth system models. Because horizontal velocities are vertically invariant within the SSA framework, 3‐D processes can be approximated locally with each material point using a series of vertical layers, and subsequently vertically integrated if needed for implementation into the next SSA solution. While the same can be applied to mesh‐based Eulerian methods, the associated advection schemes are not only dissipative, but involve solving a matrix equation for each 3‐D field that scales in computational expense with the number of layers used. However, as for 2‐D fields, horizontal advection of 3‐D fields using the MPM‐SSA framework avoids artificial diffusion and only requires explicitly updating 2‐D material point locations, rather than solving a matrix equation as in Eulerian schemes. We employ this 3‐D approach to model orthotropic damage evolution in ice shelves in Part II (Huth et al., 2021). Given the simplicity and efficiency of 3‐D advection in the MPM‐SSA framework, it may prove useful even for modeling coupled processes that do not necessarily demand error‐free advection as well, such as temperature evolution, firn compaction, fabric anisotropy, and marine ice formation. We also note that a full 3‐D implementation of material point methods for full‐Stokes models is also possible for studying individual glaciers, but it would be prohibitively expensive for continental‐scale ice sheets.

## 7 Conclusion

We presented the generalized interpolation material point method for shallow shelf ice flow, and verified that this formulation can reproduce and maintain analytical solutions for steady state ice flow and ice front advection. The advantages of this formulation include:

1. Error‐free Lagrangian advection or transport without numerical diffusion or dispersion
2. Computationally inexpensive, explicit time updates that do not require solving a matrix equation for ice thickness and history variables, such as damage. History variables may be tracked in 3‐D on a series of vertical layers associated with each material point. Horizontal advection of 3‐D fields is naturally accounted for during the 2‐D material point position update, because horizontal velocities are vertically invariant.
3. Natural tracking of the ice front and grounding line at sub‐element scale
4. Accurate schemes for boundary treatment and redistribution of thickness during particle splitting that facilitate simulations over long timescales.
5. A framework consistent with the well‐established conventions of the finite element method for shallow shelf ice flow

By choosing the particle characteristic functions to be either the Dirac delta or the “hat” functions, the present formulation can reproduce the existing implementations of sMPM and the GIMPM. We demonstrated that the sMPM shape functions are very sensitive to cell‐crossing errors and uneven distributions of material points, likely due to the quasi‐static and highly nonlocal stress regime of the SSA. By simply modifying the shape functions with a reweighting scheme in the sMPM, we can significantly decrease this sensitivity to cell‐crossing errors. This numerical error is almost entirely alleviated using the GIMPM without the reweighting scheme, so it is more appropriate for many applications on timescales of decades to centuries. However, a major advantage of the reweighted sMPM over the GIMPM is that it is applicable with adaptive and non‐uniform quadrilateral and triangular mesh discretization, which is ideal for accurately resolving grounding line dynamics. Future work is necessary to mitigate errors in the GIMPM associated with the intense distortion and gaps in the material point distribution observed in high shear regimes over long timescales. Potential solutions for this error involve developing material point reinitialization schemes, improving GIMPM domain updating schemes, and/or implementing different shape functions. In addition, future developments should focus on implementing additional physics to fully take advantage of the GIMPM‐SSA treatment of history variables; this could be particularly beneficial when parameterizing complex 3‐D processes using a series of vertical layers assigned to each material point. Thus, the GIMPM‐SSA model can potentially develop into a powerful tool for studying large‐scale, coupled ice sheet processes simultaneously, thus enabling the accurate prediction of ice sheet response to climate change and eventually global sea level rise.

## Supporting information

Figure S1

## Data Availability

The simulations in this paper can be reproduced using the experimental setups and model source code available at https://doi.org/10.5281/zenodo.4657848.
