# Supplementary material for: A Generalized Interpolation Material Point Method for Shallow Ice Shelves. 1: Shallow Shelf Approximation and Ice Thickness Evolution
Source: J Adv Model Earth Syst. 2021 Aug 24;13(8):e2020MS002277. doi: 10.1029/2020MS002277 (PMC8459298; doi:10.1029/2020MS002277)
Supplement: Supplementary file 1 — Figure S1 [file JAME-13-e2020MS002277-s001.docx]

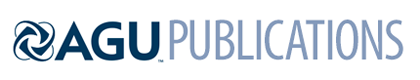


*Journal of Advances in Modeling Earth Systems*

Supporting Information for

**A generalized interpolation material point method for shallow ice shelves. Part I: shallow shelf approximation and ice thickness evolution**

A. Huth^1^, R. Duddu^2,3^, and B.E. Smith^4^

^1^Department of Earth and Space Sciences, University of Washington, Seattle, WA, USA, ^2^Department of Civil and Environmental Engineering, Vanderbilt University, Nashville, TN, USA, ^3^Department of Earth and Environmental Sciences, Vanderbilt University, Nashville, TN, USA, ^4^University of Washington, Applied Physics Laboratory, Polar Science Center, Seattle, WA, USA

**Contents of this file**

Figure S1

**Introduction**

This supporting information provides the shape functions of the standard and generalized interpolation material point methods.


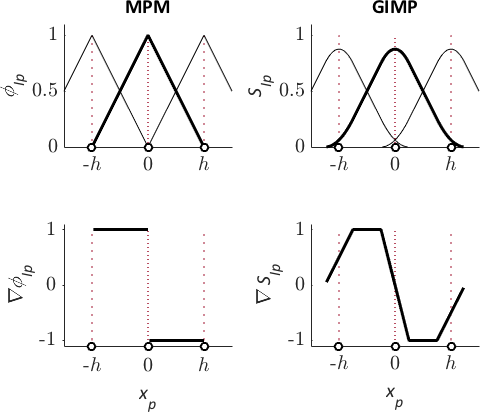


Figure S1. The 1-D sMPM (left) and GIMPM (right) shape functions for a node positioned at x_i_ = 0 with the length of an element given by *h*. The length of the material point domain used in the GIMPM convolution is *h*/2 (green shading).
